# Supplementary figures and images for: Maf/ham1-like pyrophosphatases of non-canonical nucleotides are host-specific partners of viral RNA-dependent RNA polymerases
Source: PLoS Pathog. 2022 Feb 18;18(2):e1010332. doi: 10.1371/journal.ppat.1010332 (PMC8893687; doi:10.1371/journal.ppat.1010332)

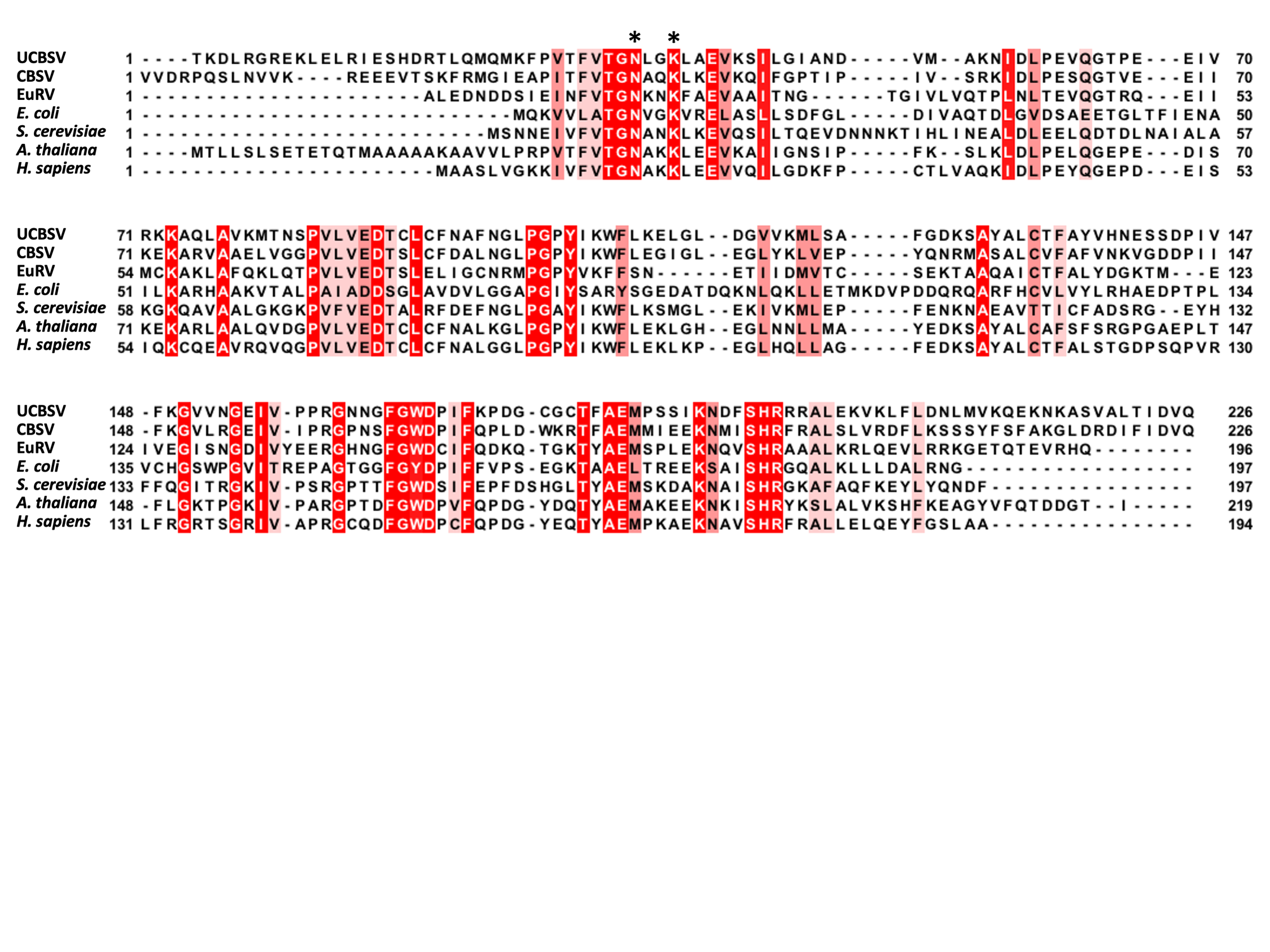

Supplement: S1 Fig — The intensity of red colour correlates with the degree of amino acid identities. Black asterisk indicates the fully-conserved K present in all known HAM1 proteins that was mutated by A in this study (see Figs 3 and S2). (TIF) [file ppat.1010332.s001.tif]

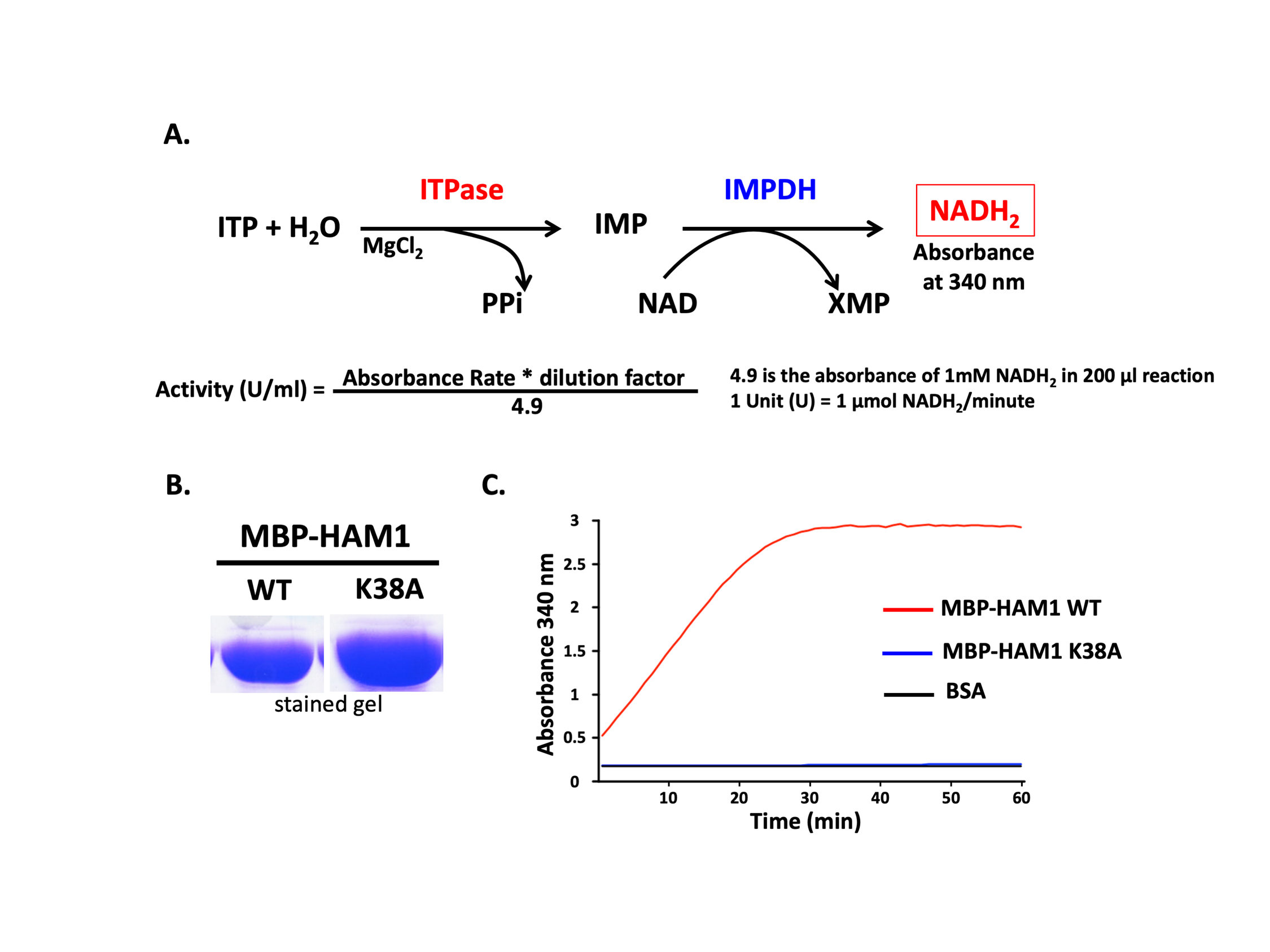

Supplement: S2 Fig — (A) Coupled reaction used to measure ITPase activity. HAM1 ITPase hydrolyses ITP in the presence of Mg+2 to produce IMP and pyrophosphate (PPi). IMP is then oxidized by inosine monophosphate dehydrogenase (IMPDH) in the presence of NAD to produce XMP and NADH2, which is directly monitored in real time at 340 nm. The ITPase activity (U/ml) can be calculated as shown. (B) Cropped polyacrylamide gel stained with coomasie blue that illustrates the yield of MBP-HAM1 in elution fractions. The indicated MBP-HAM1 versions in fraction two, which had the higher protein concentration, are shown. (C) Kinetics of ITP hydrolysis, measured by the formation of NADH2 as indicated in (A), when 1 μl of a 1:10 dilution of the elution fractions shown in (B) were used in the reaction mix. (TIF) [file ppat.1010332.s002.tif]

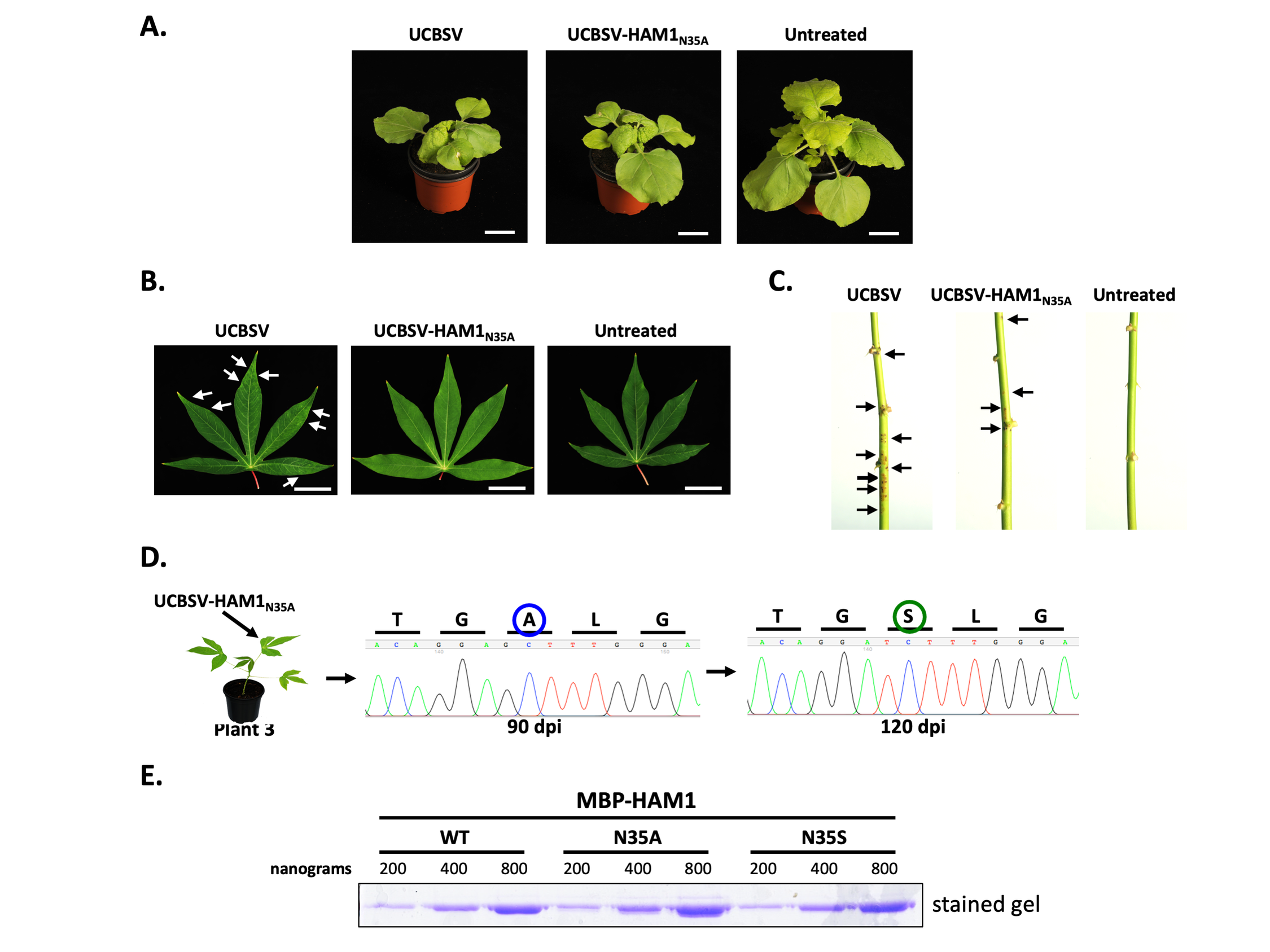

Supplement: S3 Fig — (A) Representative pictures under white light of infected and non-treated N. benthamiana plants at 11 days post-inoculation. White bar = 4 cm. (B) Representative pictures under white light of upper non-inoculated leaves, at 40 days post-inoculation (dpi), of cassava plants inoculated with the indicated viruses. White arrows indicate the presence of leaf chlorosis induced by UCBSV in cassava. White bar = 4 cm. (C) Representative pictures under white light of stems, at 90 dpi, of cassava plants inoculated with the indicated viruses. Black arrows indicate the presence of brown streaks induced by UCBSV in cassava. (D) Chromatograms of Sanger sequencing results of the DNA fragment of interest amplified by RT-PCR. RNA samples deriving from upper non-inoculated leaves of the indicated cassava plant inoculated with UCBSV-HAM1N35A were used as template. Leaves for RNA preparation were harvested at both 90 and 120 days post-infection (dpi). Coloured circles surround both the original and the naturally introduced mutation. (E) Cropped polyacrylamide gel stained with coomasie blue that shows that equivalent amounts of the indicated proteins were used for the experiment in Fig 4E. 1:20 dilutions (10, 20 and 40 nanograms, correspondingly) were used for the measurement of ITPase activity. (TIF) [file ppat.1010332.s003.tif]

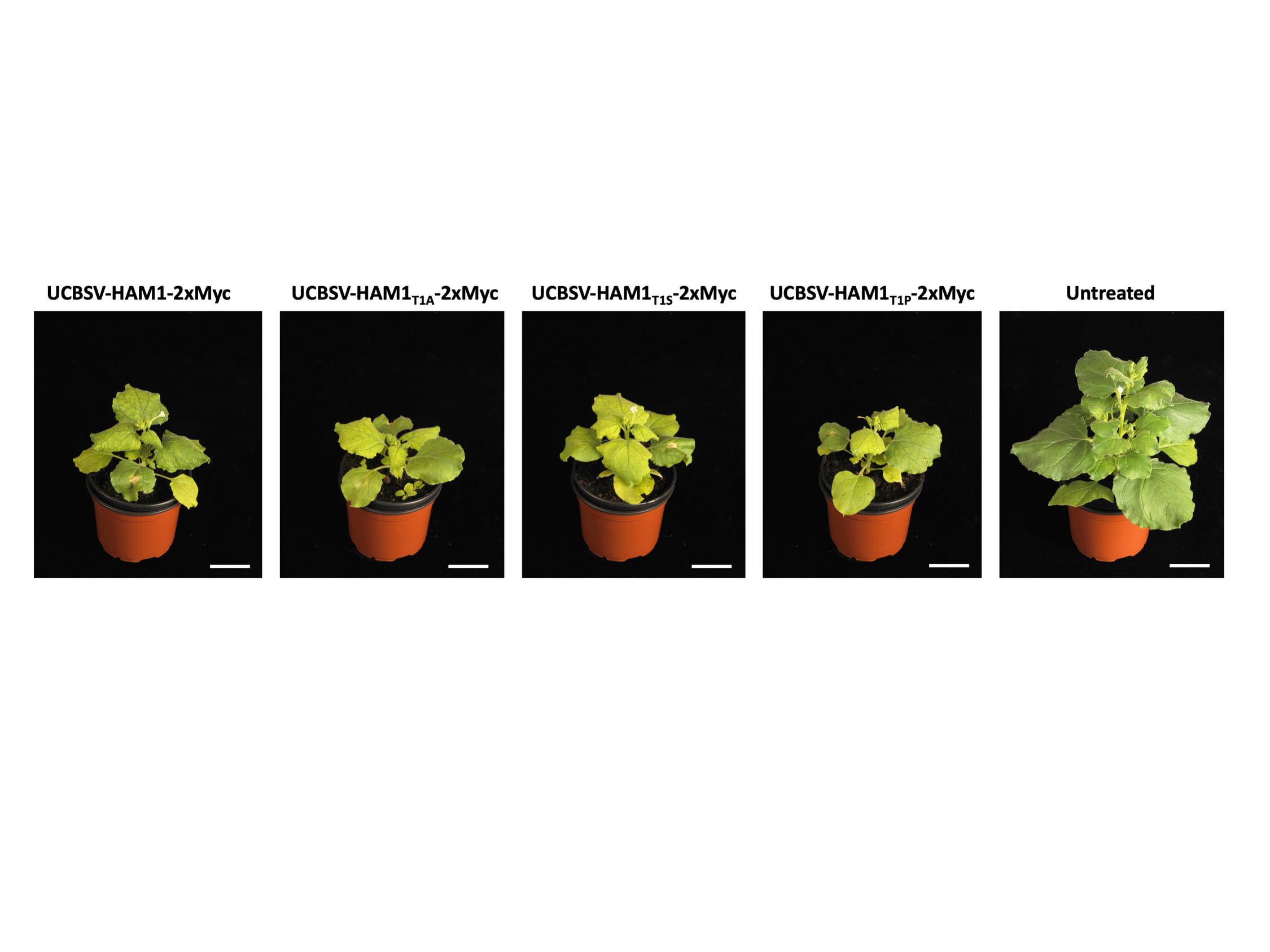

Supplement: S4 Fig — Representative pictures of infected and non-treated N. benthamiana plants taken at 12 days post-inoculation. Bar = 4 cm. This image complements the information shown in Fig 6. (TIF) [file ppat.1010332.s004.tif]

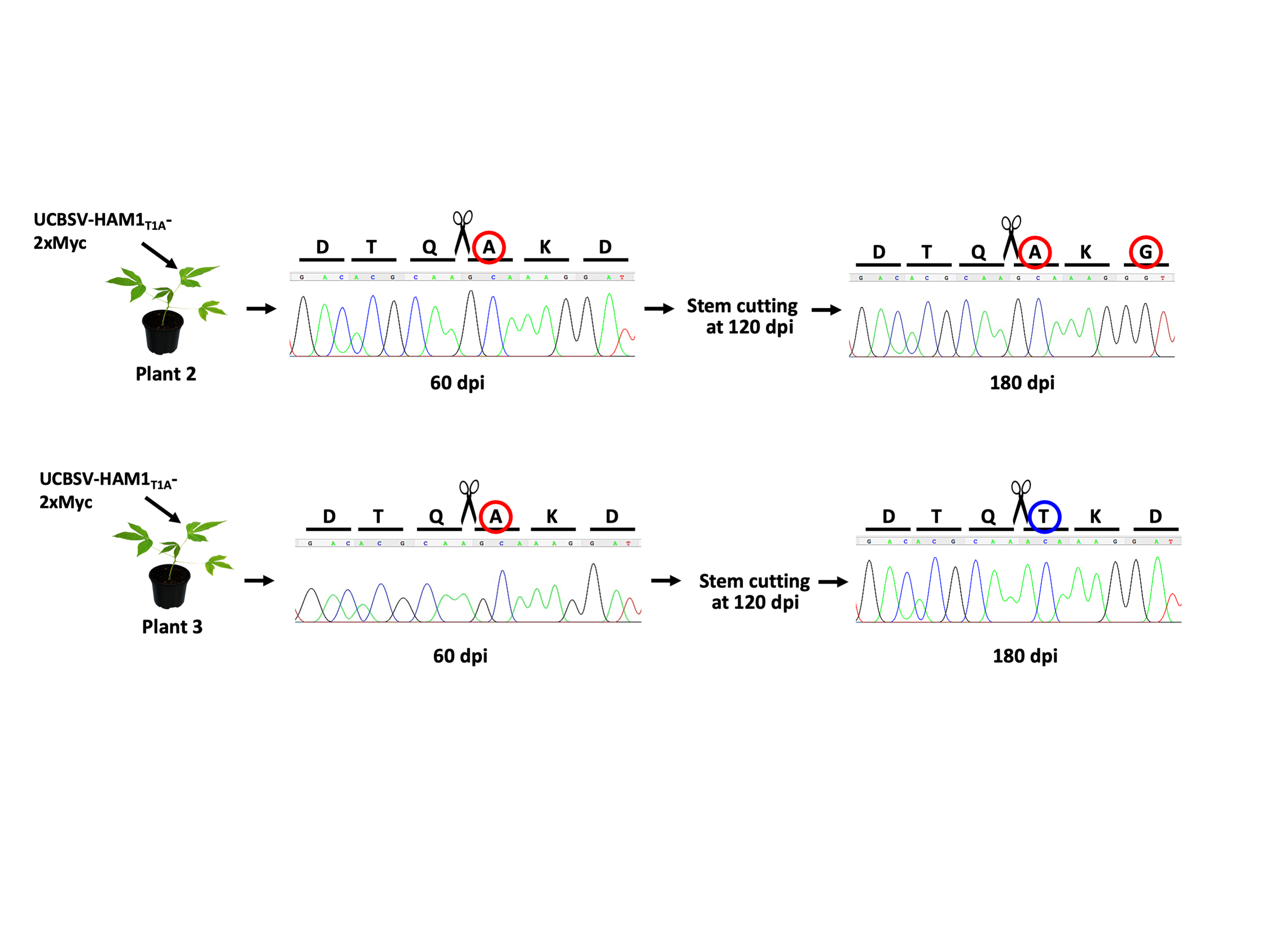

Supplement: S5 Fig — Chromatograms of Sanger sequencing results of the DNA fragment of interest amplified by RT-PCR. RNA samples obtained from upper non-inoculated leaves of cassava plants inoculated with the UCBSV-HAM1T1A-2xMyc mutant, and from plants derived from a stem cutting passage were used as template. Leaves for RNA preparation were harvested at 60- and 180-days post-infection (dpi). Residues derived from the original mutation and from the spontaneous second mutation are surrounded by a red circle. The amino acid that appears as the reversion to the wild type variant is surrounded by a blue circle. (TIF) [file ppat.1010332.s005.tif]
