# Supplementary material for: Maf/ham1-like pyrophosphatases of non-canonical nucleotides are host-specific partners of viral RNA-dependent RNA polymerases
Source: PLoS Pathog. 2022 Feb 18;18(2):e1010332. doi: 10.1371/journal.ppat.1010332 (PMC8893687; doi:10.1371/journal.ppat.1010332)
Supplement: S1 Table — (DOCX) [file ppat.1010332.s006.docx]

**Supplementary table S1**. Oligonucleotides used in this study.

| **Primer** | **Sequence (5’-3’)** |
| --- | --- |
| #3071 | ATGGGCTACAGTAAACGACAACGTCTTAAG |
| #3072 | ATGTGTTGAAAAGCATGCACTTGC |
| #3123 | ATGAATGGTGATGATTTGATCATAGCTATTAACC |
| #3124 | ATGTGACTTGTTCCTCGCCATC |
| #3127 | ATGGCGAAGCACAAGTATAACAGAGATAAG |
| #3128 | AAATGCTTGGAAGATATTGGTCTCG |
| #3129 | ATGAATGGGGATGATCTGATCCTCAATG |
| #3130 | AAGCTGACTGTGATACCTTTGGAGG |
| #3160 | AGATATCAATGAAGAATGGTTGATAGG |
| #3162 | ACCGTTCAAGTCTTCCTCGGAGATTAGCTTTTGTTCACCGTTAATTAACCCAGCCACACTTGCTTTATTCTTCTC |
| #3163 | CCTGAACGTCAATCGTTAGAGCAAGATCCTCTTCTGAAATTAATTTTTGTTCACCGTTCAAGTCTTCCTCGG |
| #3205 | CAGGAAATTTGGGAGCATTAGCAGAGGTGAAGTCTATTCTTG |
| #3206 | CACCTCTGCTAATGCTCCCAAATTTCCTGTCACAAATG |
| #3255 | TACCTGCAGATGTCGACTATC |
| #3256 | CAAAGGTAGAAGCAGAAACTTACCTGGCTTTCCGTGCATAC |
| #3257 | GTAAGTTTCTGCTTCTACCTTTG |
| #3258 | CTGCATATCAACAAATTTTGG |
| #3259 | CCAAAATTTGTTGATATGCAGGTTTTACAAATTTGTGGATCAC |
| #3260 | CAGCAGCACACCACTTTAG |
| #3261 | GACACGCAAGCAAAGGATTTGAGAGGAAGA |
| #3262 | CAAATCCTTTGCTTGCGTGTCAACATAAC |
| #3263 | GACACGCAATCAAAGGATTTGAGAGGAAGA |
| #3264 | CAAATCCTTTGATTGCGTGTCAACATAAC |
| #3265 | GACACGCAACCAAAGGATTTGAGAGGAAGA |
| #3266 | CAAATCCTTTGGTTGCGTGTCAACATAAC |
| #3312 | CGAACATTGTTATGTTGACACGCAAG |
| #3313 | CTTGCGTGTCAACATAACAATGTT |
| #3358 | GCTCCTCGCCCTTGCTCACCATGGCCTGAACGTCAATCGTTAGAGC |
| #3360 | ATGGTGAGCAAGGGCGAGGAGC |
| #3361 | CTTGCACATCGATTGTCAAGGTACCCTTGTACAGCTCGTCC |
| #3424 | TTTGTGACAGGAGCTTTGGGAAAATTAGC |
| #3425 | TAATTTTCCCAAAGCTCCTGTCACAAATGTAAC |
| #3647 | ATGGCAAAGCATAAGTATAATCGGG |
| #3648 | ACGCCGGTTTACTCAAAATGC |
| #3649 | ATGAATGGTGATGATCTTATATTGAATGC |
| #3650 | AGCTCTTTCGAAATTCTAGAGCG |
| #3857 | GGGGACCACTTTGTACAAGAAAGCTGGGTTCACTGAACGTCAATCGTTAGAGCC |
| #3858 | GGGGACAAGTTTGTACAAAAAAGCAGGCTTCACTAAGGATTTGAGAGGAAGAGAGAAG |
| #3872 | TTACATTTGTGACAGGATCTTTGGGAAAATTAGC |
| #3873 | TGCTAATTTTCCCAAAGATCCTGTCACAAATG |
