# Supplementary material for: Maf/ham1-like pyrophosphatases of non-canonical nucleotides are host-specific partners of viral RNA-dependent RNA polymerases
Source: PLoS Pathog. 2022 Feb 18;18(2):e1010332. doi: 10.1371/journal.ppat.1010332 (PMC8893687; doi:10.1371/journal.ppat.1010332)
Supplement: S2 Table — (DOCX) [file ppat.1010332.s007.docx]

**Supplementary table S2.** Templates and name of primers used for PCR amplifications during the construction of the indicated plasmids are shown.

| **Plasmid** | **Mutant** | **PCR** | **Primer Forward*** | **Primer Reverse*** | **Template** |
| --- | --- | --- | --- | --- | --- |
| pLX-UCBSVi | K38A | 1 | #3205 | #3130 | pLX-UCBSVi |
|  |  | 2 | #3160 | #3206 | pLX-UCBSVi |
|  |  | overlapped | #3160 | #3130 | PCR1 + PCR2 |
| pLX-UCBSVi | N35A | 1 | #3872 | #3130 | pLX-UCBSVi |
|  |  | 2 | #3160 | #3873 | pLX-UCBSVi |
|  |  | overlapped | #3160 | #3130 | PCR1 + PCR2 |
|  | | | | | |
| pLX-UCBSVi-HAM1-2xMyc | T1A | 1 | #3261 | #3130 | pLX-UCBSVi-HAM1-2xMyc |
|  |  | 2 | #3160 | #3262 | pLX-UCBSVi-HAM1-2xMyc |
|  |  | overlapped | #3160 | #3130 | PCR1 + PCR2 |
|  | T1S | 1 | #3263 | #3130 | pLX-UCBSVi-HAM1-2xMyc |
|  |  | 2 | #3160 | #3264 | pLX-UCBSVi-HAM1-2xMyc |
|  |  | overlapped | #3160 | #3130 | PCR1 + PCR2 |
|  | T1P | 1 | #3265 | #3130 | pLX-UCBSVi-HAM1-2xMyc |
|  |  | 2 | #3160 | #3266 | pLX-UCBSVi-HAM1-2xMyc |
|  |  | overlapped | #3160 | #3130 | PCR1 + PCR2 |

*The sequences of oligonucleotides used in this study are shown in Supplementary table S1
