# Supplementary material for: Maf/ham1-like pyrophosphatases of non-canonical nucleotides are host-specific partners of viral RNA-dependent RNA polymerases
Source: PLoS Pathog. 2022 Feb 18;18(2):e1010332. doi: 10.1371/journal.ppat.1010332 (PMC8893687; doi:10.1371/journal.ppat.1010332)
Supplement: S4 Table — (DOCX) [file ppat.1010332.s009.docx]

**Supplementary table S4.** Templates and name of primers used for PCR amplifications during the construction of the indicated plasmids are shown.

| **Plasmid** | **Virus** | **Fragment** | **Primer Forward^1^** | **Primer Reverse^1^** | **Template** |
| --- | --- | --- | --- | --- | --- |
| pENTR1A | EuRV | NIa | #3071 | #3072 | cDNA from an infected plant^2^ |
|  |  | NIb_C_-HAM1-CP_N_ | #3123 | #3124 | cDNA from an infected plant^2^ |
|  | UCBSV | NIa | #3127 | #3128 | pLX-UCBSVi |
|  |  | NIb_C_-HAM1-CP_N_ | #3129 | #3130 | pLX-UCBSVi |
|  | CBSV | NIa | #3647 | #3648 | pYES2-CBSV-F2^3^ |
|  |  | NIb_C_-HAM1-CP_N_ | #3649 | #3650 | pYES2-CBSV-F2^3^ |

^1^The sequences of oligonucleotides used in this study are shown in Supplementary table S1

^2^cDNA was prepared from RNA of *Euphorbia milii* leaves infected with EuRV (Leibniz Institute DSMZ - German Collection of Microorganisms and Cell Cultures GmbH)

^3^This plasmid harbours a cDNA segment that corresponds to the 3’half part of the CBSV genome. Kindly provided by Gary Foster.
