# Supplementary material for: Maf/ham1-like pyrophosphatases of non-canonical nucleotides are host-specific partners of viral RNA-dependent RNA polymerases
Source: PLoS Pathog. 2022 Feb 18;18(2):e1010332. doi: 10.1371/journal.ppat.1010332 (PMC8893687; doi:10.1371/journal.ppat.1010332)
Supplement: S5 Table — (DOCX) [file ppat.1010332.s010.docx]

**Supplementary table S5.** Relative concentration of the indicated nucleotides in samples from either *Nicotiana benthamiana* or *Manihot esculenta* leaves. For normalization, the average of GTP in *N. benthamiana* is equal to 1.

|  | **Samples** | **CTP** | **UTP** | **ATP** | **ITP** | **GTP** | **XTP** |
| --- | --- | --- | --- | --- | --- | --- | --- |
| ***Nicotiana benthamiana*** | **1** | 2.74 | 1.62 | 1.58 | 1.08 | 1.18 | 1.44 |
|  | **2** | 2.16 | 1.40 | 1.52 | 0.98 | 0.97 | 2.29 |
|  | **3** | 2.44 | 1.22 | 1.59 | 0.99 | 0.85 | 1.92 |
|  | **4** | 2.21 | 1.82 | 1.57 | 1.01 | 1.03 | 1.50 |
|  | **5** | 2.59 | 1.41 | 1.43 | 1.09 | 0.94 | 1.66 |
|  | **6** | 2.38 | 1.92 | 1.44 | 1.12 | 1.03 | 1.72 |
|  | **7** | 2.14 | 1.88 | 1.45 | 1.15 | 1.05 | 2.04 |
|  | **8** | 2.54 | 1.61 | 1.49 | 1.06 | 0.95 | 1.87 |
|  | **9** | 2.30 | 1.52 | 1.67 | 1.12 | 1.09 | 1.94 |
|  | **10** | 2.35 | 1.67 | 1.68 | 1.05 | 1.02 | 1.75 |
|  | **11** | 2.42 | 1.70 | 1.52 | 1.11 | 0.97 | 1.90 |
|  | **12** | 2.50 | 1.52 | 1.49 | 1.08 | 0.90 | 1.79 |
| ***Manihot esculenta*** | **1** | 2.22 | 3.03 | 1.73 | 4.02 | 1.70 | 8.81 |
|  | **2** | 2.28 | 2.92 | 1.63 | 3.75 | 1.66 | 8.50 |
|  | **3** | 2.35 | 3.62 | 1.87 | 4.09 | 1.97 | 8.43 |
|  | **4** | 2.93 | 3.22 | 1.72 | 4.01 | 2.18 | 7.59 |
|  | **5** | 2.46 | 3.37 | 1.79 | 3.58 | 1.67 | 7.19 |
|  | **6** | 2.17 | 2.81 | 1.84 | 4.22 | 1.78 | 8.68 |
|  | **7** | 2.30 | 2.67 | 1.89 | 4.00 | 1.89 | 7.74 |
|  | **8** | 2.24 | 3.74 | 2.00 | 4.30 | 1.94 | 8.23 |
|  | **9** | 2.69 | 3.46 | 1.73 | 3.45 | 1.99 | 8.00 |
|  | **10** | 2.24 | 3.32 | 1.90 | 3.91 | 1.78 | 8.21 |
|  | **11** | 2.62 | 3.12 | 1.75 | 3.99 | 1.82 | 8.13 |
|  | **12** | 2.41 | 3.21 | 1.83 | 3.87 | 1.90 | 8.11 |
