# Supplementary material for: Maf/ham1-like pyrophosphatases of non-canonical nucleotides are host-specific partners of viral RNA-dependent RNA polymerases
Source: PLoS Pathog. 2022 Feb 18;18(2):e1010332. doi: 10.1371/journal.ppat.1010332 (PMC8893687; doi:10.1371/journal.ppat.1010332)
Supplement: Attachment — Submitted filename: 117252_1_rebuttal_2146485_qxjnpw.docx [file ppat.1010332.s011.docx]

**Manuscript number:** RC-2021-00826

**Corresponding author(s):** Adrian A., Valli

## 1. General Statements

***This section is optional. Insert here any general statements you wish to make about the goal of the study or about the reviews.***

Our manuscript aimed to understand the meaning and role of maf/ham1-like pyrophosphatases (HAM1) in RNA viruses. By studying the only known RNA viruses described so far that express HAM1, we demonstrated for the first time that a protein of this type works in partnership with viral RNA-dependent RNA replicases, as they are covalently bound during the infection. In addition, we found strong evidences that the presence of HAM1 in the virus is due to the high levels of non-canonical nucleotides in their natural hosts. These results are the first ones supporting the idea that host non-canonical nucleotides play a role in antiviral defense, something considered by other scientists but not yet published (e.g., https://www.gu.se/om-universitetet/hitta-person/martinlagging “inhibition of ITPase may point to novel antiviral and antibacterial strategies”).

As observed in reviewer’s comments (included below), one of the experts (reviewer 2) is very positive regarding the manuscript, whereas the other expert (reviewer 1) is positive with the first part of the manuscript (host-specific requirement of viral HAM1) but shows some concerns with the second part (levels of non-canonical nucleotides in different hosts and the existence of a replicase-Ham1 partnership). It is critical here to highlight that those concerns of reviewer 1 are founded, in our opinion, on a few unfortunate misunderstandings.

First, the reviewer 1 opens the possibility that the high levels of non-canonical nucleotides are “the cause but not the reason”. The experiment shown in Figure 4 is a comparison of NTP levels in two viral hosts, one in which viral HAM1 is required for the infection and one in which viral HAM1 is not required for the infection. Importantly, these two hosts are not infected in this experiment. Then, differences in NTP levels can never be the cause of an infection.

Finally, reviewer 1 is not convinced about “the possible direct interaction of Ham1-Nib”, and because of that she/he asks for more experiments on the matter. It is relevant here to mention that we have never used the word “interaction” in the manuscript, as we mainly refer to NIb-HAM1 as a partnership. Indeed, our experiments demonstrate, by using several approaches, that NIb and HAM1 remain covalently bound during viral infection.

A more extensive point-by-point response to comments from reviewer 1 are shown below.

## 2. Point-by-point description of the revisions

***This section is mandatory.* *Please insert a point-by-point reply describing the revisions that were already carried out and included in the transferred manuscript.***

**Reviewer #1**

**Reviewer #1 (Evidence, reproducibility and clarity (Required)):**

**A very limited number of plant virus genomes, such as cassava brown streak virus (CBSV), USBSV and Euphoriba ringspot virus (EuRSV) have acquired the inosine triphosphatase (ITPase) belonging to the Ham1 enymze family. The ms by Adrian Valli et al. described that the enzyme activity of Ham1 of UCBSV is only essentailly for its infectivity in its natural host cassava, but not in the experimental host Nicotiana benthamiana. They also provided some evidences that Ham1 of UCBSV maybe invovled in interaction with the viral RdRP-NIb. Also They provided some evidence to suggest that the concentration difference of non-canonical nucleotides in cassava could explain the functional role of Ham1 in its natural host cassava. It is a nice report to increase our knowledge to help our understanding in viral Ham1. However the major conclusions of this version is overclaimed largerly. the first conclusion is OK. The other two needs more evidences. The concentration of non-canonical nucleotides need extensive repeats and time-course data. Also how about the situations in virus-infected plants compared with mock control. They may also consider other possibility of Ham1 in plant defense response and signalling pathways. For me, the symptom of cassava brown streak and laso EuRSV is more likely with defensive response. Virus-induced necrosis observed in natural host cassava and other Euphorbiaceous plant is associated iwth pathways invovled in the hypersensitive response. Mostlikely, the level of ITP/XTP they called at high level is the cause, but not the reason. Second, the possible direct interaction of Ham1-NIb need further experimental data of protein-protein interaction. So far they only provided one set of data. They should have at least two additional methods to prove its direction interaction. Meanwhile the major part of Abstract is overclaimed.**

Responses:

First of all, we would like to thank this reviewer for her/his time and kind comments.

We do not agree with the reviewer when she/he states that “the major conclusions of this version is overclaimed largerly (*sic*)”, mainly because we believe that this comment is based on a few unfortunate misunderstandings. Here we try to clarify two points with the aim of addressing the reviewer’s concerns:

1- The experiment shown in Figure 4 is a comparison of NTP levels in two viral hosts, one in which viral HAM1 is required for the infection and one in which viral HAM1 is not required for the infection. Importantly, analyzed samples come from not infected plants; then, differences in NTP levels can never be the consequence of the infection, as the reviewer suggests. We are currently doing experiments aiming to explain why cassava plants have a much higher levels of non-canonical nucleotides than *Nicotiana benthamiana*, and we have found that the reason relies on the subcellular localization of the machinery in charge of degrading endogenous non-canonical nucleotides (these results will be included in a coming manuscript). We find very interesting the reviewer suggestion regarding the analysis of NTPs in infected plants. We will hopefully do this analysis soon.
2- The reviewer is not convinced about NIb and HAM1 interaction. First of all, we would like to state that we have never used the word “interaction” in the manuscript, as we mainly refer to NIb-HAM1 as a partnership. Indeed, our experiments show, by different approaches, the existence of HAM1 covalently linked to NIb and the relevance of the unprocessed NIb-HAM1 product during viral infection. These approaches are:

-Biochemistry: by tagging HAM1 with an epitope we were able to detect, mainly, a protein with the expected size for NIb-HAM1 fusion protein.

-Experimental evolution: by directed mutation we got viral versions in which both proteins are expressed separately. In further infections this mutant naturally evolved two independent mutations, which recovered the wild type phenotype: bound NIb-HAM1.

-Partnership conservation: in the three viruses of the *Potyviridae* family expressing HAM1, this protein is mainly produced as a fusion protein with the viral RdRP.

**Reviewer #1 (Significance (Required)):**

**It helps to enrich our understanding in plant virology area.**

Response:

We do not agree with the reviewer statement that appears to limit the impact of our work to plant virologists. As the reviewer 2 indicates (see below), our work is relevant for “Virologists, evolutionary biologists, geneticists”. The fact that we report for the first time (i) a partnership between a viral RdRP and a pyrophosphatase in RNA viruses and (ii) the importance of host non-canonical nucleotides as antivirals, means that our results will be significant for more areas than just plant virology.

**Reviewer #2**

**Reviewer #2 (Evidence, reproducibility and clarity (Required)):**

****Summary:****

**Manuscript RC-2021-00826 describes how the Maf/ham1-like amino acid sequence encoded by Uganda cassava brown streak virus (UCBSV) is indispensable for successful infection of cassava but not of the model host Nicotiana benthamiana. Authors used an infectious UCBSV clone in a reverse genetics approach to elegantly highlight why the virus genome has incorporated a host sequence during its evolution. The Maf/ham1/RNA-dependent RNA polymerase (RdRP) intermediary of UCBSV polyprotein proteolytic processing increases virus fitness compared with mature proteins, suggesting that the Maf-ham1-like protein is functional when covalently linked to the viral RdRP. The role of Maf/ham 1 in UCBSV could be potentially attributed to an intrinsic high concentration of noncanonical nucleotides in cassava, as suggested by comparative HPLC-MS/MS analyses. This study provides new and exciting insights into the functional implication of the viral Maf/ham1 protein in the biology of some of the viruses that infect cassava.**

****Major comments:****

**The conclusions are justified and convincing. No additional experiments are required to support of the claims of the manuscript. Methodologies are well described and reproducible by others. There is no issue with the reproducibility of the experiments and soundness of the data.**

Response:

We would like to thank the reviewer for her/his kind and positive words.

****Minor comments:****

**The manuscript is well written, and the figures are clear and accurate. Prior studies are referenced appropriately. The following few minor editorial changes could be considered to improve the overall quality of the text:**

Response:

Again, we would like to thank the reviewer for her/his kind, positive words and corrections. All these changes were considered and introduced in a new version of the manuscript.

**Line 37: Change for to to**

Done.

**Lines 39-40: Eliminate in nature**

Done.

**Line 44: ... the ITPase activity for infectivity in ...**

Done.

**Line 46: Change show to showed**

Done.

**Line 48: Change indicate to indicated**

Done.

**Line 55: Would versatile rather than inventive be more appropriate here?**

Inventive was replaced by versatile

**Line 59: italicize Potyviridae**

Done.

**Lines 65 and 70: Change Potyvirids to Potyviruses, as potyvirids is not a term recognized by the ICTV**

We normally prefer to use the word Potyvirids, instead of Potyviruses, when referring to all members of the *Potyviridae* family independently of their genera (as explained in the main text). The idea is avoiding confusions, as Potyviruses might also refer particularly to members of the genus *Potyvirus* in the *Potyviridae* family. We and others have been using the term Potyvirids in the past even though, as the reviewer well states, it is not recognized by the ICTV.

**Line 81: Change into to in**

Done.

**Line 85: Change dangerous to detrimental**

Done.

**Line 89: Change separated to distinct**

Done.

**Line 123: Eliminate further**

Done.

**Line 128: Change illumination to light**

Done.

**Line 218: ... and the sap was finger-rubbed onto two ...**

Done.

**Line 241: Ponceau red was sued to verify ...**

Done.

**Line 247: ... was verified by electrophoresis in agarose gel ...**

Done.

**Line 251 ... the cDNAs were used as template ...**

Done.

**Line 286: Change retardation to delay**

Done.

**Lines 307-308: Was the virus progeny tested in the infected plants rather than those obtained after passages?**

The viral progeny in the primary infected plants (plants inoculated with the infectious clone) were not sequenced as it produced a main DNA fragment after RT-PCR amplification with the size that corresponds to the entire unmodified virus. As shown in Figure 1D, DNA fragments of smaller sizes were also produced, but they did not accumulate as much as the DNA that corresponds to the unmodified virus. In the end, this result might indicate that some deletions already originated in the primary infected plants. In any case, it does not change the interpretation and the relevance of results obtained after one passage, which is the result extensively explain in the main text.

**Line 316: Change aspect to type**

Done.

**Lines 350, 480, and 482: Change potyvirids to potyviruses**

This change was not introduced. We prefer to keep the term potyvirids to refer to members of the whole *Potyviridae* family (reasons are explained above).

**Line 351: Italicize Arabidopsis**

Done.

**Line 373: eliminate about**

Done.

**Line 381: Change respect to relative**

Done.

**Lines 397-398: ... x 7 cleavage sites (Adams et al., 2005). Curiously, ...**

Done.

**Line 401: ... tobacco etch virus, another potyvirus, showed that S x T mutation...**

Done.

**Lines 402-403: ... Figure 4D in Dougherty et al., 1988)**

Done.

**Line 409: ... as control, were inoculated ...**

Done.

**Line 412: Change indicating to indicated**

Done.

**Lines 416-417: ... expected size for the sole ...**

Done.

**Line 425: ... cleavage sites ...**

Done.

**Lines 442-443: ... Figure 6B). Moreover, Sanger sequencing ...**

Done.

**Line 444: Change 2 to two**

Done.

**Line 470: ... showed that the T1A/D3G ...**

Done.

**Line 415: Change helpful to essential**

Done.

**Line 561: Change excited to exciting**

Done.

**Reviewer #2 (Significance (Required)):**

**Significance of the advance: The research is novel and transformative with regards to the evolutionary biology of plant viruses. It clearly documents why how and why some viruses need to incorporate a plant protein into their genome to establish infection of certain hosts.**

Response:

We thank the reviewer for this comment. We would like to add that our findings are transformative not only with regards to the evolutionary biology of plant viruses, but also with that of viruses independently of their hosts, and discloses a first hint on how potential mechanistic differences in the processing of non-canonical nucleotides can provide hosts with advantages in terms of resistance to pathogens.

**Place the work in the context of the existing literature. This study sheds light on the role of the viral Maf/ham1 protein in the biology of some of the viruses that infect cassava. This is an area of research that was not previously addressed with such a depth and rigor. The research is novel and transformative with regards to the evolutionary biology of plant viruses.**

Response:

Once again, we thank the reviewer for this comment. As stated just above, we think that our findings are transformative not only with regards to the evolutionary biology of plant viruses, but also with pathogen-host interactions in general.

**State what audience might be interested in and influenced by the reported findings: Virologists, evolutionary biologists, geneticists**

Response:

We thank the reviewer for this comment. Indeed, we also believe that the audience that might be interested in our manuscript is not only that interested on just plant viruses.

**Define your field of expertise with a few keywords to help the authors contextualize your point of view. Plant viruses, virus-host interactions, virus-vector interactions, management**

****Referee Cross-commenting****

**Some of the criticisms such as the repetition of experiments to measure the concentration of non-canonical nucleotides in virus-infected plants are well taken. Other criticisms seem speculative but should help authors revise the text.**

Response:

A following manuscript will contain experiments aiming to understand why and how Euphorbiaceous plants have high levels of non-canonical nucleotides. Indeed, experiments comparing the level of NTPs in different plant compartments and at different infection stages are ongoing.
