# Supplementary material for: Maf/ham1-like pyrophosphatases of non-canonical nucleotides are host-specific partners of viral RNA-dependent RNA polymerases
Source: PLoS Pathog. 2022 Feb 18;18(2):e1010332. doi: 10.1371/journal.ppat.1010332 (PMC8893687; doi:10.1371/journal.ppat.1010332)
Supplement: Attachment — Submitted filename: Point-by-Point_Responses.docx [file ppat.1010332.s012.docx]

**Point-by-point responses to Editor and reviewers’ comments**

***Editor:***

***Thank you very much for submitting your manuscript "Maf/ham1-like pyrophosphatases of non-canonical nucleotides are host-specific partners of viral RNA-dependent RNA polymerases" for consideration at PLOS Pathogens. As with all papers reviewed by the journal, your manuscript was reviewed by members of the editorial board and by several independent reviewers. In light of the reviews (below this email), we would like to invite the resubmission of a significantly-revised version that takes into account the reviewers' comments. Especially the authors need to address the reviewer 1's major concern, i.e., lack of direct evidence of viral inhibition by ITP/XTP. The reviewer 1 also suggested some feasible experiments. We are expecting that the authors can address it timely using these suggested experiments or alternative experiments the authors deem appropriately.***

***We cannot make any decision about publication until we have seen the revised manuscript and your response to the reviewers' comments. Your revised manuscript is also likely to be sent to reviewers for further evaluation.***

**Response:**

Here we are submitting a significantly-revised version of our manuscript, in which we have included (i) one more figure (Figure 4 in the current version), (ii) two more Supplementary figures (S2 and S3 in the current version), (iii) one more Supplementary table (S5 in the current version), as well as (iv) all the required changes indicated/suggested by reviewers.

We would like to highlight that the new data mainly aim to respond the reviewer 1’s major concerns, as indicated by the Editor in his decision letter.

Below, point-by-point responses to reviewers’ comments are included.

***Part I - Summary***

***Please use this section to discuss strengths/weaknesses of study, novelty/significance, general execution and scholarship.***

***Reviewer #1: This manuscript presents a few interesting observations and the authors provided working model, but the conclusions remain largely descriptive without plant host direct genetic evidences. Without evidences based on these mutant plant materials invovled in ITP and XTP biosynthesis, the authors claim a not directly demonstrated conception about UCBSV requires HAM1 to infect Cassava due to elevated levels of non-canonical nucleotides. Second, there is no virus infectivity difference for these UCBSV-HAM1 mutants. Therefore there is no direct relationship between its enzyme activity (HAM1) with virus infection.***

**Response:**

We would like to thank the reviewer 1 for the time she/he used to profoundly evaluate our manuscript. Indeed, all her/his comments have been taken into account to strongly improve the manuscript (see below).

Regarding the first point made by the reviewer here, we totally agree with her/him in the importance of having host direct genetic evidences about the involvement of non-canonical nucleotides on viral defence. As the reviewer know, having stably silenced/CRISPRed plants in order to knock down/out genes of interest is a time-consuming task. Indeed, we are working on that, and do anticipate that the newly generated data on these regards will set the bases of another manuscript.

Regarding the second point, it is critical to highlight that the reviewer seems to misunderstand the previously presented data. The reviewer states that “***there is no virus infectivity difference for these UCBSV-HAM1 mutants”,*** which is not correct. In the former manuscript, we had included two mutants affected in HAM1 and, consequently, in non-canonical nucleotides hydrolysis: a complete HAM1 deletion (UCBSV-ΔHAM1) and the K38A point mutation (UCBSV-HAM1_K38A_), which was expected to destroy the HAM1 pyrophosphatase catalytic center. As the reviewer can see in our former and current manuscript (Figure 2 and 3 in both cases), these two mutant viruses, which infected *N. benthamiana* (low concentration of XTP/ITP) as well as the wild type virus, were unable to infect cassava (high concentration of XTP/ITP). Therefore, the reviewer’s statement: “***there is no direct relationship between its enzyme activity (HAM1) with virus infection”*** is wrong.

In any case, given the reviewer’s concern on this regard, and in order to make our case even stronger, we have carried out a few new experiments:

1- HAM1_K38A_, which was unable to support UCBSV infection in cassava, was expressed in bacteria and purified. As expected, it resulted to be a pyrophosphatase knock-out mutant. This result complements former Figure 3 and are included as Supplementary Figure (S2 in the current version).
2- Another UCBSV mutant virus, UCBSV-HAM1_N35A_, was built. On basis of published experiments in *Escherichia coli* (Savchenko *et al.,* 2007), HAM1_N35A_ (N10 in the HAM1 from *E. coli*) was expected to have reduced pyrophosphatase activity. UCBSV-HAM1_N35A_ resulted to have lower fitness than the wild type virus in cassava. Moreover, this mutant evolved during the course of infection to UCBSV-HAM1_N35S_ (serine at position 35) in two out of three plants. If HAM1 pyrophophatase activity were critical for the virus to infect cassava, then we would expect that the naturally evolved HAM1_N35S_ have higher activity than HAM1_N35A_. To test this, we expressed and purified these two mutants and demonstrated that our prediction was true. These new data are included in the current manuscript in the new Figure 3 and new Supplementary Figure S3.

All in all, we can conclude that, indeed, there is a direct and perfect correlation between the pyrophosphatase activity on non-canonical nucleotides of HAM1 and the capacity of UCBSV to infect its natural host.

***Reviewer #2: In this manuscript, using an infectious cDNA clone and reverse genetics approach, the authors found that the Ugandan cassava brown streak virus (UCBSV)-encoding Maf/ham1-like protein is functional when covalently linked to the viral RdRP for adaptation to its natural host plant cassava, in which over-accumulation of non-canonical nucleotides (ITP/XTP) was detected by HPLCMS/MS experiments. This study is novel and provides exciting insights into the evolutionary biology of plant viruses, under the high concentration of non-canonical nucleotides pressure inside the host, by incorporating an ITP/XTP pyrophosphatase into RdRP to support successful replication and infection.***

**Response:**

We would like to thank the reviewer 2 for her/his time, and for her/his positive words about our study.

***Part II – Major Issues: Key Experiments Required for Acceptance***

***Please use this section to detail the key new experiments or modifications of existing experiments that should be absolutely required to validate study conclusions.***

***Generally, there should be no more than 3 such required experiments or major modifications for a "Major Revision" recommendation. If more than 3 experiments are necessary to validate the study conclusions, then you are encouraged to recommend "Reject".***

***Reviewer #1: 1. In the response letter page 1, the author claimed that "These results are the first ones supporting the idea***

***that host non-canonical nucleotides play a role in antiviral defense, something considered by other scientists***

***but not yet published (e.g., https://www.gu.se/om-universitetet/hittaperson/martinlagging***

***“inhibition of ITPase may point to novel antiviral and antibacterial strategies”). Again the authors claimed that this host-specific constraint is due to an unexpected high concentration of non-canonical nucleotides in cassava but lower ITP and XTP levels in Nicotiana benthamiana. Could you please provide the original set of data for each of 12 samples? Why no statitic analysis for Fig. 4? Also some host genetic manipulation experiments could be provided more direct evidences on this points by silencing some genes invovled in ITP and XTP biosynthesis. Also with these plant genetic manipulation data such as VIGS or CRISPR/CAS9 genome editing will provide a direct evidence to show it is a novel host target for antivirals.***

**Response:**

We would like to thank reviewer 1 for these interesting comments.

As requested, we have included the original data set for each of 12 samples in the new Supplementary table S5. In addition, we have complemented the former Figure 3 (Figure 4 in the current version) with a bar graph that clearly illustrates the data presented in Supplementary table S5. Also, as requested by reviewer 1, we have included a statistical analysis of these data to show those differentially significant differences in NTP accumulation in *N. benthamiana* and cassava.

Indeed, the idea of using VIGS and CRISPR to manipulate genes involved in non-canonical nucleotide syntheses, as stated in our previous response to reviewer 1 (see above), is on its way. Unfortunately, getting data from these experiments will take a rather long time.

***Reviewer #2: The conclusions are appropriate and well supported by the experiments. This reviewer also appreciate that authors have made efforts to address issues in their revision.***

**Response:**

Once again, we would like to thank the reviewer 2. Indeed, all her/his previous comments were taken into account in order to improve our manuscript.

***Part III – Minor Issues: Editorial and Data Presentation Modifications***

***Please use this section for editorial suggestions as well as relatively minor modifications of existing data that would enhance clarity.***

***Reviewer #1: 1. please add Potyvirues before RNA-dependent RNA polymerases in the title.***

***2. please remove the words such as “Ebola of plants” in the abstract.***

***3. In the abstract and main text of the manuscript, please consider to replace the word "unexpected high concentration in non-canonical nucleotides in cassava", which is***

1- We would prefer to keep the title as in the former manuscript. Adding the word *Potyviruses* will give readers the wrong idea that a partnership between a viral RNA-dependent RNA polymerase and HAM1 has been described in the past for other viruses, and that our case is just another. Importantly, as far as we know, this is the first time than this partnership is shown, and as such, we think the former title better illustrates our finding.

2- Although it is true that we do not know who coined the term *“Ebola of plants”* to refer to the disease caused by UCBSV and CBSV, we did find a register of such a term as late as in 2017 in a report from the prestigious news channel CNN:

https://edition.cnn.com/2017/07/11/africa/cassava-disease-east-africa/index.html

If possible, we would prefer to maintain in the manuscript this alternative name for the disease caused by Ipomoviruses in cassava, as it gives a clear idea to the general public about the importance of these viruses for food security (mainly in Africa).

3- Unfortunately, this reviewer’s comment is not complete, so it is not possible for us to see the reason behind her/his recommendation regarding the replacement of ***"unexpected high concentration in non-canonical nucleotides in cassava"*** in the text. To make our statement clear, we would like to mention that the concentration of non-canonical nucleotides in any living organism is expected to be low in order to avoid their potential deleterious effects (as explained in the manuscript). The fact that cassava plants accumulate high concentration of ITP/XTP is, therefore, “unexpected”. In fact, in one of our on-going studies we have uncovered the reason underpinning this phenomenon in plants from the *Euphorbiaceae* family. These particular results will be included in a future report.

**Reviewer #2: (No Response)**
